# Gross Hematuria Revealing Bilateral Renal Metastases From Pulmonary Squamous Cell Carcinoma: A Case Report

**DOI:** 10.7759/cureus.114128

**Published:** 2026-08-07

**Authors:** Charaf Eddine Harchaoui, Mohamed Amine Harchaoui, Mohamed Tetou, Mohammed Mrabti, Youness Boukhlifi, Abdessamad El Bahri, Ahmed Ameur

**Affiliations:** 1 Urology, Mohammed V Military Hospital, Rabat, MAR; 2 Urology, Faculty of Medicine, Hassan II University of Casablanca, Casablanca, MAR; 3 Urology, Faculty of Medicine, Mohammed V University of Rabat, Rabat, MAR

**Keywords:** bilateral renal metastases, gross hematuria, pulmonary squamous cell carcinoma, renal biopsy, secondary renal tumors

## Abstract

Renal metastases from lung cancer are uncommon in clinical practice and are usually asymptomatic. Bilateral renal metastases presenting with gross hematuria as the initial manifestation of pulmonary squamous cell carcinoma (SCC) are exceptionally rare. We describe the case of a 67-year-old man with a 50-year smoking history who presented with painless hematuria, bilateral flank pain, intermittent fever, and weight loss. Abdominal computed tomography (CT) revealed bilaterally enlarged kidneys containing multiple hypodense lesions, while chest CT revealed a right hilar mass with collapse of the right middle and lower lobes. CT-guided biopsies of both the renal and pulmonary lesions confirmed a poorly to moderately differentiated SCC. Immunohistochemistry showed positivity for p63, CK5/6, and CK7 and negativity for CK20 and uroplakin, confirming bilateral renal metastases from a primary pulmonary SCC. Following multidisciplinary discussion, systemic therapy was initiated, as surgical management was not indicated. This case highlights the importance of including metastatic lung cancer in the differential diagnosis of bilateral renal masses presenting with hematuria and underscores the pivotal role of renal biopsy in establishing the correct diagnosis.

## Introduction

Lung carcinoma remains the leading cause of cancer-related mortality worldwide, largely because of its high metastatic potential and the frequent presence of advanced disease at diagnosis [1,2].

In patients with non-small cell lung cancer, metastatic spread most frequently involves the brain, skeleton, liver, and adrenal glands, whereas renal involvement represents an uncommon site of dissemination [1,3]. Although autopsy studies have reported renal metastases in up to 19% of patients who died from lung cancer, these lesions are typically asymptomatic and therefore frequently remain undetected during life. As a result, renal metastases are only rarely identified at the time of the initial diagnosis of lung cancer [1].

Hematuria, whether microscopic or macroscopic, can result from a variety of causes ranging from benign infections or kidney stones to serious urological neoplasms. In adults, the presence of gross hematuria must always raise suspicion of a malignant tumor of the urinary tract until proven otherwise [4].

In our case, hematuria served as an unusual presenting symptom that led to the diagnosis of primary lung cancer, an exceptionally rare presentation.

## Case presentation

A 67-year-old man with no significant past medical history presented with a one-month history of bilateral flank pain, intermittent fever, and hematuria. He additionally reported fatigue and an unintentional weight loss of 10 kg over the preceding two months. He had no history of tuberculosis or prior antituberculous treatment and had a smoking history of more than 50 pack-years. He reported no history of alcohol consumption, occupational or environmental toxic exposure, or a family history of similar malignancies. Physical examination was unremarkable.

**Figure 1:**
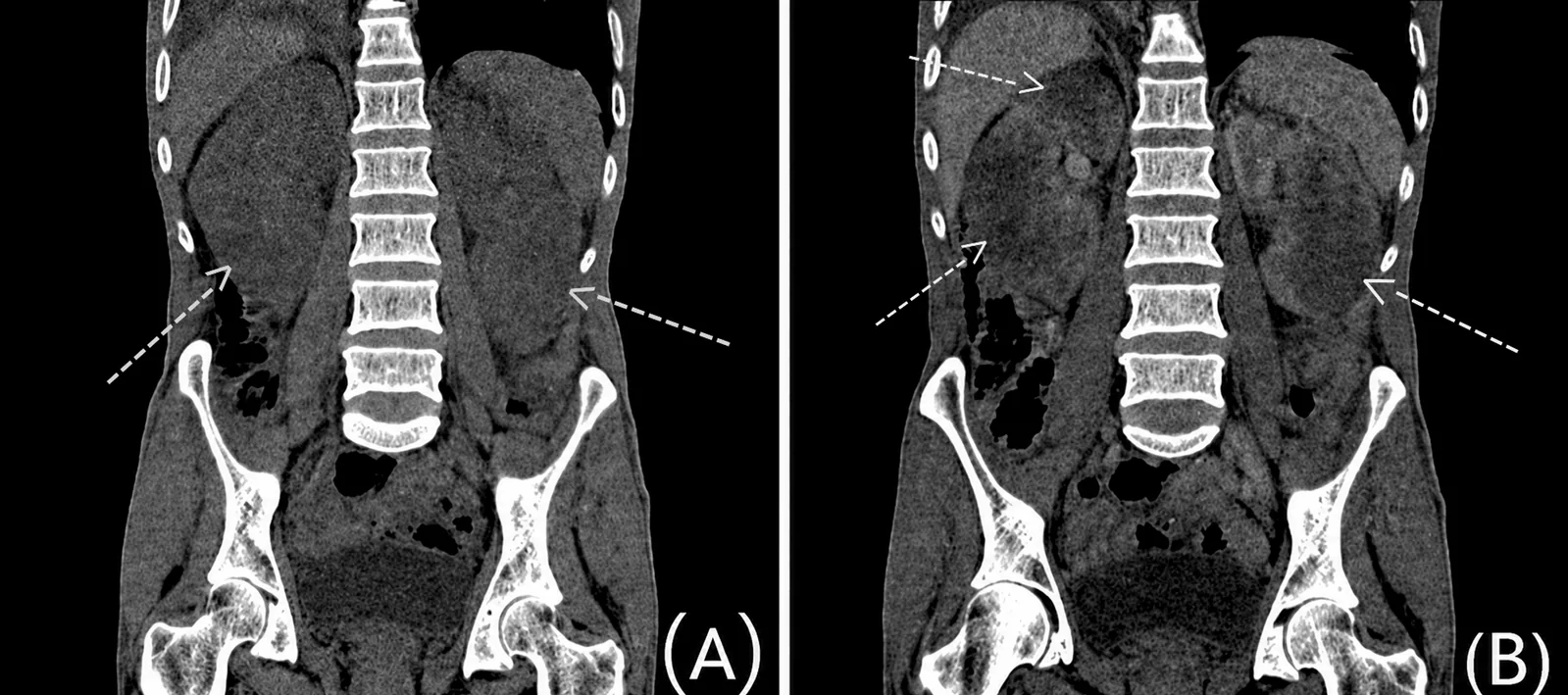
Contrast-enhanced abdominal computed tomography showing bilaterally enlarged kidneys with multiple well-defined hypodense lesions: (A) unenhanced scan and (B) contrast-enhanced scan

Contrast-enhanced abdominal computed tomography (CT) revealed bilaterally enlarged kidneys containing multiple well-defined hypodense lesions (Figure 1).

**Figure 2:**
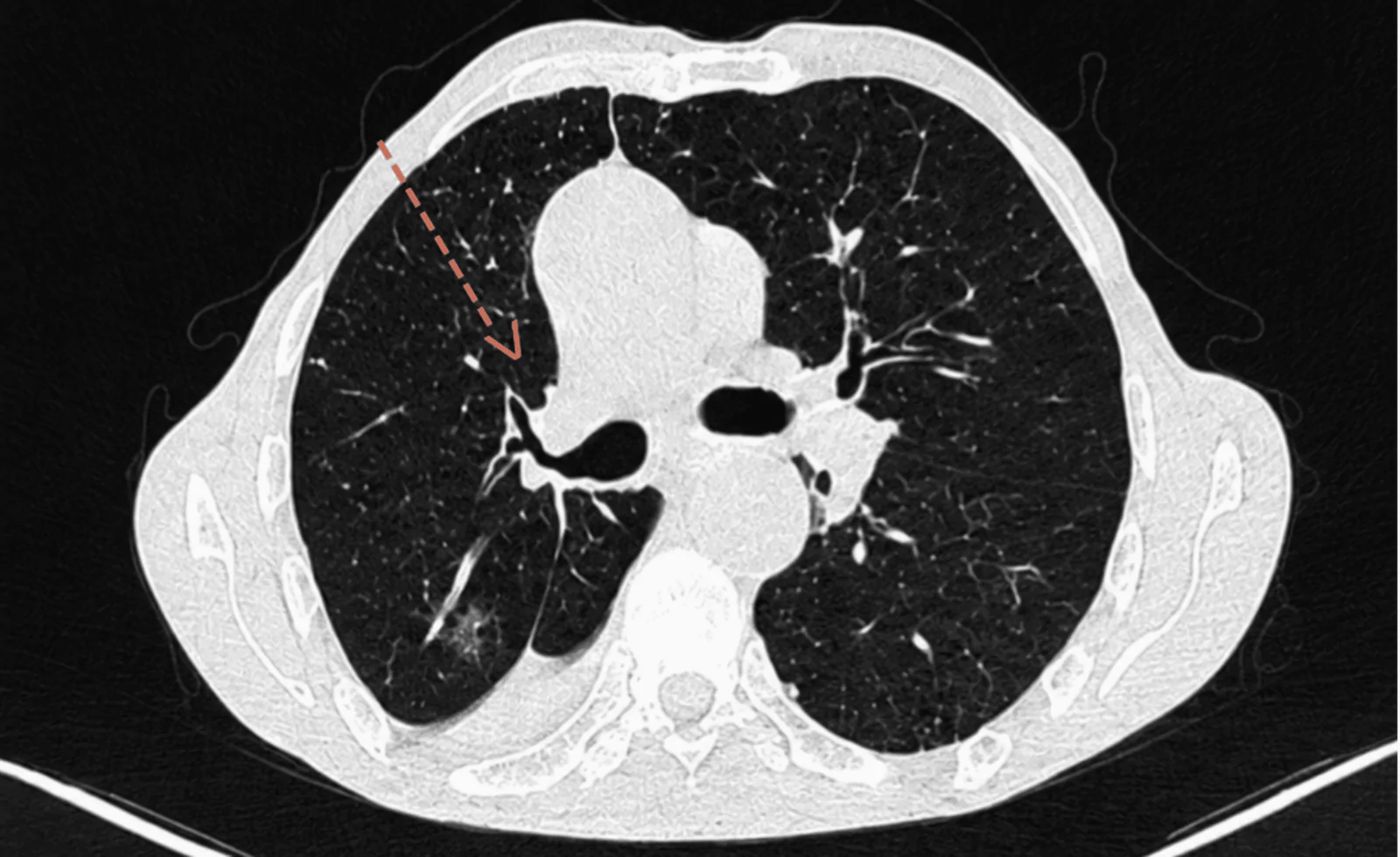
Contrast-enhanced chest computed tomography demonstrating a right hilar mass obstructing the bronchus intermedius

Subsequent chest CT revealed a right hilar mass obstructing the bronchus intermedius, resulting in the collapse of the right middle and lower lobes (Figure 2).

The patient subsequently underwent CT-guided percutaneous core needle biopsies of both the renal and pulmonary lesions. Histopathological examination revealed a poorly to moderately differentiated squamous cell carcinoma (SCC). Immunohistochemical analysis demonstrated positivity for p63, CK5/6, and CK7, with negative staining for CK20 and uroplakin, confirming metastatic pulmonary SCC.

Following discussion at the multidisciplinary tumor board, the decision was to proceed with systemic therapy, as there was no indication for surgical intervention.

## Discussion

Despite advances in diagnosis and systemic therapy, lung cancer continues to account for the highest number of cancer-related deaths worldwide. Non-small cell lung cancer accounts for approximately 85% of cases, with SCC and adenocarcinoma being the predominant histological types, representing approximately 30% and 50% of diagnoses, respectively. SCC is known to primarily metastasize to regional lymph nodes, as well as to the bones, liver, and central nervous system [2].

The development of renal metastases in lung cancer usually signifies disseminated disease and is associated with limited survival and an unfavorable prognosis [5]. Most patients with renal metastases from lung carcinoma also have concurrent metastatic involvement of other organs, particularly the liver, bones, and adrenal glands. In a series of 146 patients with lung carcinoma, renal metastases accounted for only 1.4% of all metastatic sites [6].

Renal metastases usually remain clinically silent and are frequently detected incidentally during imaging performed for staging. It is estimated that microscopic hematuria occurs in no more than one-third of patients [7]. This rarity is due to the fact that metastatic deposits tend to localize in the renal cortex, near the glomerular vascular plexus, thus often sparing the urothelium [8]. The most common presenting symptom associated with renal metastases was flank pain (30%), followed by hematuria (16%), weight loss, night sweats, and fever [7].

Differentiating renal metastases from a synchronous primary renal malignancy can be challenging, as imaging findings are often nonspecific. When a second primary renal tumor is suspected, histopathological confirmation is essential to establish the diagnosis [9].

In our patient, the presence of hematuria initially raised the suspicion of a primary renal neoplasm; however, histopathological examination of the renal biopsy confirmed metastatic SCC. Nevertheless, the renal metastatic lesion imaging characteristics may resemble those of primary tumors.

## Conclusions

Bilateral renal metastases from pulmonary SCC presenting with gross hematuria are exceedingly rare. This case emphasizes that metastatic disease should be considered in patients presenting with bilateral renal masses and hematuria, particularly in smokers. Histopathological confirmation by renal biopsy is essential to establish the diagnosis and guide appropriate oncological management while avoiding unnecessary surgical intervention.
